# A single night light exposure acutely alters hormonal and metabolic responses in healthy participants

**DOI:** 10.1530/EC-16-0097

**Published:** 2017-01-25

**Authors:** Mohammed S Albreiki, Benita Middleton, Shelagh M Hampton

**Affiliations:** Department of Biochemistry and PhysiologyCentre for Chronobiology, Faculty of Health and Medical Sciences, University of Surrey, Guildford, Surrey, UK

**Keywords:** metabolism, melatonin, light at night, endogenous response

## Abstract

Many animal studies have reported an association between melatonin suppression and the disturbance of metabolic responses; yet, few human studies have investigated bright light effects on metabolic and hormonal responses at night. This study investigated the impact of light on plasma hormones and metabolites prior to, and after, an evening meal in healthy participants. Seventeen healthy participants, 8 females (22.2 ± 2.59 years, mean ± s.d.) and 9 males (22.8 ± 3.5 years) were randomised to a two-way cross-over design protocol; dim light (DL) (<5 lux) and bright light (BL) (>500 lux) sessions, separated by at least seven days. Saliva and plasma samples were collected prior to and after a standard evening meal at specific intervals. Plasma non-esterified fatty acid (NEFA) levels were significantly higher pre-meal in DL compared to BL (*P* < 0.01). Plasma glucose and insulin levels were significantly greater post-meal in the BL compared to DL session (*P* = 0.02, *P* = 0.001), respectively. Salivary melatonin levels were significantly higher in the DL compared to those in BL session (*P* = 0.005). BL at night was associated with significant increases in plasma glucose and insulin suggestive of glucose intolerance and insulin insensitivity. Raised pre-prandial NEFA levels may be due to changes in insulin sensitivity or the presence of melatonin and/or light at night. Plasma triglyceride (TAG) levels were the same in both sessions. These results may explain some of the health issues reported in shift workers; however, further studies are needed to elucidate the cause of these metabolic changes.

## Introduction

Artificial light exposure at night has become commonplace throughout the developed world (1, 2). Light has been linked to various complex mechanisms such as the synchronisation of the circadian system (3). Circadian rhythms are seen in any biological processes that display an endogenous oscillation of about 24 h. They are generated by the suprachiasmatic nuclei (SCN) located in the anterior hypothalamus and influenced by external cues called zeitgebers (commonly daylight). Melatonin is considered the classical phase marker for assessing the timing of the mammalian biological clock. The SCN drives the daily rhythms in hormone concentrations such as insulin, glucagon, corticosterone (4, 5, 6) and enzymes involved in lipid and glucose metabolism, such as glucose-6-phosphate dehydrogenase (7, 8). Therefore, disruption of circadian coordination may be manifested by endocrine imbalances (9), incidence of obesity (10) and type 2 diabetes (11, 12). This raises a controversial issue as to whether aberrant light exposure may influence metabolism by changing the time of the circadian system (13). It has been reported that blood glucose increases during light exposure and decreases during darkness in rats (14, 15). Others have reported the melatonin-induced inhibition of insulin secretion via cyclic adenosine monophosphate (cAMP) and cyclic guanine monophosphate (cGMP), and the presence of melatonin receptors 1 (MT1) and 2 (MT2) in pancreatic tissues of both rats and humans (16). Additionally, acute melatonin administration in healthy women has been reported to impaired glucose tolerance in both the morning and evening (17). The impact of melatonin administration on lipid metabolism has been demonstrated in experimental animals (18, 19) and humans (20, 21). In addition, melatonin has been reported to influence insulin (16) and glucagon (22), which in turn affect enzymes involved in lipid metabolism such as hormone-sensitive lipase (HSL) and lipoprotein lipase (LPL).

A majority of the previous studies investigating the effect of light on hormone and metabolic responses have either been carried out on experimental animals (14) or under-restricted conditions in humans such as constant routine (16, 23, 24) including the administration of exogenous melatonin (17, 21). The aim of this study was to investigate the impact of broad spectrum bright light exposure (>500 lux) on healthy young participants prior to and after a late-evening meal. The hypothesis being that a single night of light exposure would be associated with changes in glucose tolerance, insulin sensitivity and lipid profiles. The findings could have health implications for individuals with a nocturnal lifestyle including nightshift work.

## Participants and methods

### Recruitment

All procedures received a favourable ethical opinion from the University of Surrey Ethics Committee (UEC/2013/93/FHMS) and were conducted in accordance with the Declaration of Helsinki (1975) as revised in 1983 and conformed to international ethical standards. Volunteer information was coded and held securely in compliance with the Data Protection Act, UK (1998). All participants gave written informed consent after full explanation of the purpose and nature of all procedures involved.

### Participants and screening

Seventeen healthy participants, 8 females (22.2 ± 2.59 years; mean ± s.d.) and 9 males (22.8 ± 3.5 years) were recruited from students and staff at the University of Surrey. The two genders were matched for age and body mass index (BMI) (Table 1). Participants were all non-smokers and taking no medication except for mild analgesics. All females were on oral contraceptive pills. Participants had not crossed more than two time zones and/or worked night shifts during the month before the study. All participants completed screening questionnaires including Pittsburgh Sleep Quality Index (PSQI), Horne-Östberg (HÖ) and Munich chronotype.

**Table 1:** Participant demographics.

	**Male** (*n* = 9)	**Female** (*n* = 8)	**P** (M vs F)
Age (year)	22.3 ± 3.6	22.6 ± 2.2	0.84
Body weight (kg)	68.4 ± 7.9	63.8 ± 8.5	0.26
Height (m)	1.8 ± 1.7	1.7 ± 0.1	0.14
BMI (kg/m^2^)	22.9 ± 2.5	22.7 ± 2.2	0.88
Caffeine (week)	10.1 ± 6.2	11.4 ± 9.1	0.74
Alcohol (week)	2.8 ± 2.3	3.8 ± 4.2	0.56
PSQI^a^	3.3 ± 1.2	4.1 ± 1.5	0.24
HÖ^a^	51.2 ± 8.1	52.4 ± 10.9	0.81
MCTQ^a^ (h)	4.7 ± 1.2	4.9 ± 1.1	0.87

Values are mean ± s.d., *P* values were calculated by 2-tailed unpaired test.

a Values given are those obtained during the screening session.

BMI, body mass index; HÖ, Horne–Östberg questionnaire; MCTQ, Munich Chronotype Questionnaire; PSQI, Pittsburgh Sleep Quality Index.

### Pre-laboratory measurements

All participants maintained a standard self-selected regular sleep–wake cycle (nocturnal sleep duration of 6.5–8 h, with sleep onset between 23:00 h and 01:00 h) for at least 7 days before the in-laboratory sessions, as confirmed by actigraphy (AWL, Cambridge Neurotechnology, UK) and sleep diaries (Table 2). 24-h prior to the laboratory sessions, participants were required to refrain from caffeinated drinks, alcohol, excessive exercise and medicine intake. In addition, participants performed a 48-h sequential urine collection to measure 6-sulfatoxymelatonin (αMT6s), the major urinary metabolite of melatonin, via radioimmunoassay (Stockgrand Ltd., University of Surrey, Guildford, UK). The acrophase of 6-sulfatoxymelatonin was determined by cosinor analysis, enabling meal intakes (supper) to be individually scheduled to occur on the rising phase of each participants’ endogenous melatonin rhythm.

**Table 2:** Screening sleep and basal hormone and metabolite data.

	**BL**	**DL**	***P***
Sleep start^a^ (h:min)	23:54 ± 00:14	00:18 ± 00:14	0.1
Sleep end^a^ (h:min)	07:21 ± 00:10	07:35 ± 00:09	0.1
Sleep duration^a^ (h)	06:37 ± 00:16	06:24 ± 00:14	0.5
% Sleep efficiency^a^	81.9 ± 2.8	78.06 ± 3.5	0.4
Sleep latency^a^ (h:min)	00:35 ± 00:12	00:48 ± 00:12	0.5
Fragmentation index^a^	26.4 ± 2.9	28.81 ± 3.4	0.5
Basal glucose^b^ (mmol/L)	4.9 ± 0.3	4.8 ± 0.2	0.7
Basal insulin^b^ (pmol/L)	107 ± 34	105 ± 28	0.9
Basal NEFAs^b^ (mmol/L)	0.75 ± 0.1	0.91 ± 0.1	0.07
Basal TAGs^b^ (mmol/L)	1.1 ± 0.1	1.2 ± 0.2	0.4
Basal melatonin^b^ (pg/mL)	1.8 ± 0.4	2.6 ± 0.6	0.05

Values are mean ± s.e.m. Sleep parameters *n* = 15 hormone and metabolic basal data *n* = 17.

a Values are obtained from 7 days prior to BL and DL sessions. ^b^Values represented the basal samples (*T* = −360 min) from each clinical session. Sleep parameters were analysed based on data obtained from sleep diaries and Actiwatch data. *P < 0.05.

### Laboratory session

**Figure 1:**
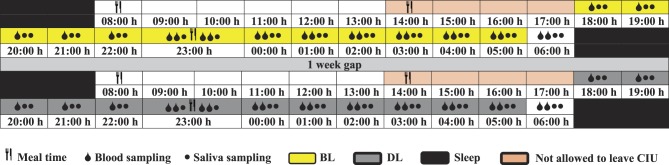
Study protocol of BL and DL sessions. The schematic figure represents the study protocol for a participant with plasma melatonin onset at 22:30 h. All interventions (see key) were relative to each participants’ melatonin onset.

All participants were randomised to a two-way cross-over design protocol; dim light and bright light. All study sessions were held at the Clinical Investigation Unit (CIU), which was equipped with overhead light control. During the dim light session, lighting levels were <5 lux and in the bright light session, lighting was >500 lux between 18:00 h and 06:00 h the next day (Fig. 1). Participants were randomly coded alphanumerically divided into groups A and B using the sealed envelope method. Group A attended BL session and then the DL session, whereas group B completed the sessions in reverse order. Participants were kept awake and semi-recumbent throughout the study session, except during visits to the toilet. A set breakfast was provided at 08:00 h, whereas lunch and supper (test meal) times were individualised on the basis of the acrophase time of urinary aMT6s. The fasting period between lunch and supper was 9–10 h. Participants consumed an isocaloric and non-carbonated evening meal at a time estimated to be within 30 min of endogenous melatonin onset (1066 kcal, 38 g protein, 104 g CHO, 54 g fat, 7 g fibre) (Table 3).

**Table 3:** Carbohydrate, protein, fat, fibre and energy for each of the meals and overall composition of all three meals.

Meal (g)	**Energy** (kcal)	**Protein** (g)	**CHO** (g)	**Fat** (g)	**Fibre** (g)
Breakfast	627	15	98	16	14
Lunch	927	25	115	38	19
Test meal ‘supper’	1066	38	104	54	7
Total	2620	78	317	105	40
% composition*		15%	59%	19%	7%

* Percentages were calculated proportionally from the total daily consumption.

Blood samples were collected hourly from 18:00 h until the evening meal; then, every 15 min for the first hour after the meal, then at 30-min intervals until the end of the session. In total, 22 blood samples were collected in each session from each participant for analysis of insulin, glucose, triglyceride (TAG) and non-esterified fatty acids (NEFAs). Saliva samples were collected every 30 min from 18:00 h to 06:00 h the following day for analysis of melatonin levels.

### Light measurements

Light intensity was measured at 2 different positions horizontal level (direction of gaze) (*n* = 221; DL 1.06 ± 0.06 lux, BL 305 ± 10.1 lux; mean ± s.e.m.) and vertical level towards the lights (*n* = 221; DL 1.21 ± 0.13, BL 552.7 ± 16; mean ± s.e.m.).

**Figure 2:**
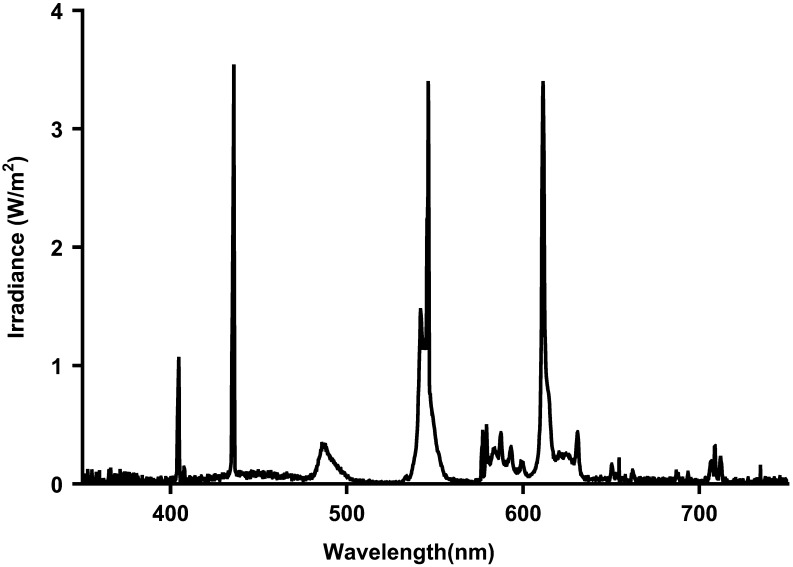
Fluorescent light composition.

Spectral composition of the light source was measured using a R203 power radiometer at horizontal (DL 0.001 w/m^2^, BL 0.98 w/m^2^) and vertical level (DL 0.0008 w/m^2^, BL 0.73 w/m^2^). The light source provided in both studies are fluorescent light, and the spectral composition of the light is shown in Fig. 2.

### Assay procedures

Plasma glucose, TAG (Werfen Ltd, Warrington, UK) and NEFAs (Randox Laboratories Ltd, Crumlin, UK) were analysed by standard automated enzymatic spectrophotometric methods (ILAB600). The interassay coefficients of variation were less than 5% for glucose, NEFAs and TAGs. Plasma immunoreactive insulin was measured using radioimmunoassay (RIA) (Millipore). Salivary melatonin and urinary aMT6s were analysed using in-house RIAs (25, 26). The interassay coefficients of variation were less than 10% for melatonin (control 1 = 6.5 pg/mL (6.7%), control 2 = 24.5 (6.7%) and control 3 = 49.6 pg/mL (6.8%)) and insulin (control 1 = 100.3 pmol/L (10%), and control 2 = 332.2 pmol/L (9.5%)).

### Measurement of insulin resistance

An index of fasting insulin resistance (HOMA-IR) and postprandial insulin resistance (HOMA-PP) were determined for the evening meal in both BL and DL sessions.

HOMA-IR was calculated using the HOMA calculator based on HOMA model 2 developed by Jonathan Levy (27).

HOMA-PP was calculated as the incremental area under the curve (IAUC) glucose (mmol/L min) × IAUC insulin (U/L min).

This equation has been validated against the intravenous glucose tolerance (28).

### Data and statistical analysis

A power calculation was performed using PS software (Vanderbilt University, Nashville, Tennessee, USA) with a power of 80% and a significance level 0.05 utilising NEFA data obtained from a previous pilot investigation. From this power calculation, ≥18 participants were required.

Urine aMT6s data were subjected to cosinor analysis (Dr D S Minors at the University of Manchester, UK), to ascertain calculated peak time of aMT6s (acrophase).

All data were checked for normality using D’Agostino Pearson omnibus normality test (Graphpad). The mean value plus the standard deviation (s.d.) and standard error of mean (s.e.m.) were calculated from individual data sets. All hormonal and metabolic data were subjected to three-factor repeated measures ANOVA (condition, gender and time) followed by Tukey’s honest significance *post hoc* test to locate individual differences, using Statistica Statsoft (Tulsa, OK, USA). The trapezoidal rule was used to determine the total area under curve (TAUC). All hormone and metabolite data were analysed using TAUC, followed by 2-tailed paired Student’s *t*-test. The significance level was set at *P* < 0.05.

## Results

### Comparison of male and female participants

The mean age, body weight, height and BMI of 9 males and 8 females in this study were matched (Table 1). Caffeine and alcohol consumption over the two weeks prior to the study were not significantly different between male and female groups. Both genders were classified as neither morning nor evening types by the HÖ, and all reported a good sleep quality over a month prior to the study using the PSQI. Sleep parameters screened prior to BL and DL sessions are given in Table 2. Participants reported no significant difference in sleep prior to BL and DL sessions. No differences were observed in hormone and metabolic concentrations at the start of the each study session (Table 2).

### Plasma levels prior to the test meal (*T* = 0)

**Figure 3:**
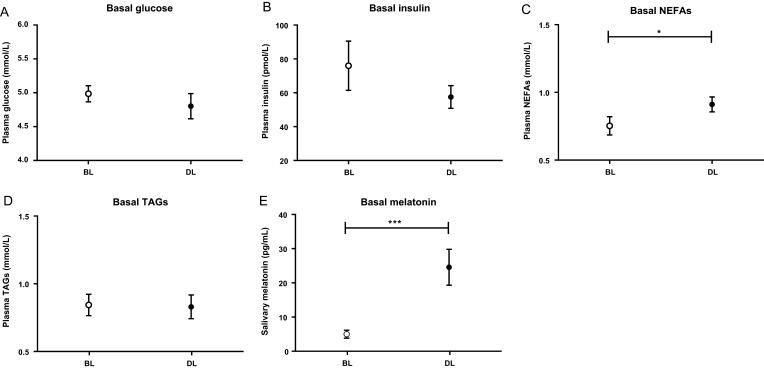
Plasma glucose (A), insulin (B), NEFAs (C), TAGs (D) and melatonin (E) levels (mean ± s.e.m.) prior to the test meal at time = 0 in all participants (*n* = 17) during DL () and BL () sessions. *P < 0.05, and ***P < 0.001.

Basal levels of plasma insulin, glucose, TAGs and NEFAs from samples collected immediately prior to the meal (*T* = 0) are shown in Fig. 3. Basal glucose and insulin showed no significant differences between BL and DL sessions. Basal NEFAs were significantly higher in DL than those in BL (*P* = 0.02). No significant difference was seen in basal TAGs between DL and BL (*P* = 0.81). Basal melatonin levels were significantly greater in DL than those in BL (*P* < 0.001) (Fig. 2).

### Hormone and metabolic responses prior to the meal (T-360-0)

**Figure 4:**
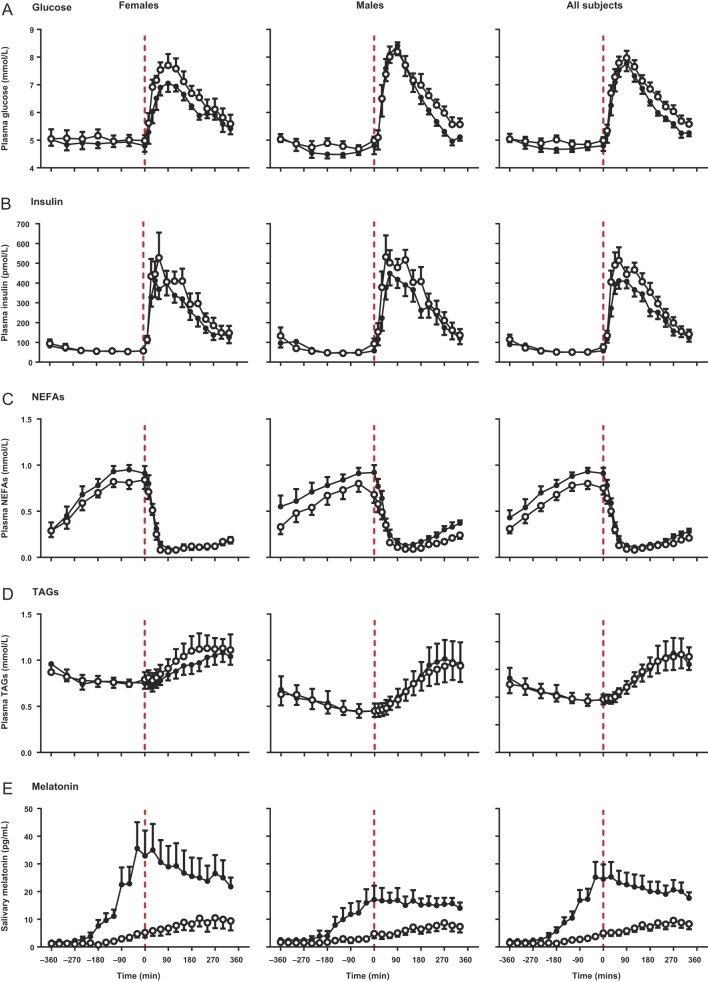
Plasma glucose (A), insulin (B), NEFAs (C), TAGS (D) and salivary melatonin (E) (mean ± s.e.m.) levels prior to and after a standard evening meal (time = 0 red dotted line) during DL () and BL () sessions in males (*n* = 9), females (*n* = 8) and all participants (*n* = 17).

There were no significant differences between BL and DL sessions in plasma glucose, insulin and TAGs concentrations prior to evening meal. Pre-evening meal NEFAs showed a significant increase in DL compared to that in BL session (*P* = 0.03). Similarly, salivary melatonin was significantly higher in the DL session (*P* < 0.001) (Fig. 4).

### Postprandial hormone and metabolic responses

Postprandial plasma glucose and insulin responses to the test meal showed a significant increase in BL compared to those in DL (*P* = 0.01, *P* = 0.008), respectively. Salivary melatonin was significantly greater in DL than that in BL sessions (*P* < 0.001). There were no differences in postprandial TAGs responses after BL and DL sessions (Fig. 3).

### Pre-prandial and postprandial hormone and metabolic responses (*T* = −360 to *T* = +330)

Graphical representations of the female, male and all participants during DL and BL sessions are shown in Fig. 3. Plasma insulin levels were significantly greater in BL than those in DL sessions (*P* = 0.001). *Post hoc* tests showed significant differences at +180, +210 and +270 min. Similarly, plasma glucose showed a significant increase in BL compared to that in DL sessions (*P* = 0.02). *Post hoc* test showed significant differences at +180 and +210 min after the meal. In contrast, there was a significant pre-prandial increase of plasma NEFA in DL session (*P* = 0.005). *Post hoc* tests showed the difference was directly prior to the evening meal (*T* = 0). Plasma TAGs showed no significant difference between the DL and BL sessions. All 4 plasma parameters showed significant effects of time, whereas no significant effects of gender were observed.

Salivary melatonin levels were significantly greater in DL than those in BL session (*P* < 0.001). *Post hoc* tests showed significant differences at pre-prandial and postprandial time points between −120 and +330 min. Both males and females show similar increase in salivary melatonin in DL compared to those in BL sessions, females showed higher levels of salivary melatonin than males although not significant (Fig. 4).

### Total area under the curve

**Figure 5:**
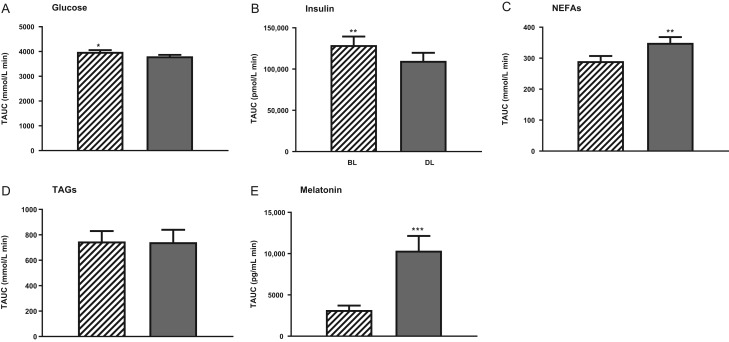
Total area under the curve (TAUCs) for glucose (A), insulin (B), NEFAs (C), TAGs (D) and melatonin (E) (mean ± s.e.m.) during BL and DL ▬ sessions in all participant (*n* = 17). *P < 0.05, **P < 0.01.

TAUCs for, insulin, glucose, NEFAs, TAGs and melatonin were calculated and are shown in Fig. 5. TAUCs for NEFAs showed a significant reduction in BL (290 ± 17 mmol/L min) compared to those in DL (350 ± 18 mmol/L min) sessions (*P* = 0.009). In contrast, a significant increase in TAUCs for plasma insulin and glucose were shown in BL compared to DL (*P* = 0.004 and *P* = 0.03), respectively. TAUC of plasma insulin was 129,119 ± 10,343 pmol/L min in DL and 109,875 ± 9817 pmol/L min BL, whereas TAUCs of plasma glucose were 3805.76 ± 60 mmol/L min in DL and 3985 ± 73 mmol/L min in BL. No significant difference was observed in plasma TAGs responses. TAUCs showed a significant suppression of salivary melatonin in BL compared to those in DL sessions (*P* < 0.0001), and TAUCs were 3171 ± 530 pg/mL min in BL session and 10,362 ± 1777 pg/mL min in DL sessions.

### HOMA-PP and HOMA-IR

**Figure 6:**
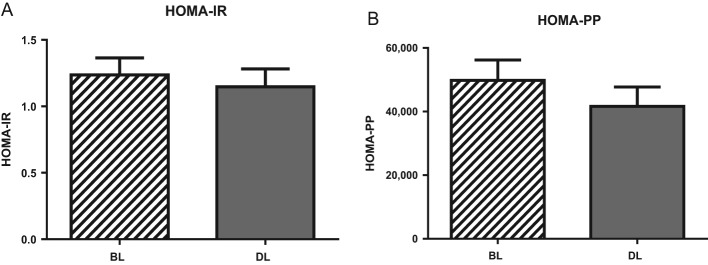
HOMA-IR (A) and HOMA-PP (B) (mean ± s.e.m.) during BL and DL ▬ sessions (*n* = 17).

HOMA-PP and HOMA-IR are shown in Fig. 6. HOMA-PP was greater but not significant in BL (49,802 ± 6428) than that in DL (41,607 ± 6141) session. Similarly, HOMA-IR was higher, yet not significantly, in BL (1.2 ± 0.1) compared to that in DL (1.1 ± 0.1) sessions.

## Discussion

Few studies have investigated the influence of light at night on plasma hormones and metabolites in healthy humans as a majority of the studies reported to have either been carried out on experimental animals (29) or under-restricted conditions in humans such as constant routine (16, 23, 30) or involved in the administration of exogenous melatonin (18, 20). To the author’s knowledge, this is the first study to investigate bright light exposure at night on hormonal and metabolic responses prior to and after a standard evening meal in healthy young individuals by exposing them to two light sessions: bright (>500 lux), equivalent to light the intensity in the workplace, and dim light (<5 lux) equivalent to candle light.

The salivary melatonin profile was significantly reduced by bright light exposure at night. This was expected as the light intensity delivered at the angle of gaze was 305 lux, and previous human studies have shown that 200 lux was sufficient to suppress salivary melatonin by 50% in healthy participants (31). In this study, a reduction of 62% in melatonin amplitude was observed in the BL session. The evening meal was targeted between 30 min and 1 h after estimated melatonin onset in both light sessions to ensure the presence of endogenous melatonin at the meal time. All participants showed the presence of endogenous melatonin at evening meal time in DL session. Our results indicate that salivary melatonin levels appear higher, but not statistically significant, in females than those in males under both light sessions agreeing with previously published research (32).

Plasma glucose responses were significantly elevated three hours after the meal in the BL session, confirmed by TAUC analysis. Insulin levels were significantly elevated after the meal in BL compared to those in the DL sessions. Raised glucose and insulin responses suggest changes in glucose tolerance and insulin sensitivity. Calculation of HOMA-PP and HOMA-IR confirmed these findings although significance was not achieved. These results agree with rodent studies that showed decreased blood glucose and insulin in constant darkness (14, 15). Fonken and coworkers observed that male mice in constant light increased body mass with reduced glucose tolerance compared to those in a standard light/darkness cycle with similar food intake (33). It is important to note that rodents are nocturnal, whereas humans are diurnal mammals with reversed rest activity cycles and hormonal rhythms, including those important in metabolic regulation, except for melatonin, which peaks during the darkness phase in both (34). Our results are also in agreement with recent evidence observed in sleep deprivation (23, 35, 36) and circadian misalignment (37) such as in jet lag and shift work, which have shown an increase of postprandial glucose and insulin that denotes insulin insensitivity and failure of beta cell compensation (38). Reduced insulin sensitivity reported in sleep debt could be due to an altered balance between the sympathetic (SNS) and parasympathetic nervous system (PNS) (35, 38), which may influence beta cell activity indirectly via cortisol and epinephrine (35, 39). High night-time cortisol has been reported to be associated with sleep loss and insulin resistance (40). Overstimulation of the SNS as a result of the wake-promoting factor orexin (36) has been reported to result in increased glucose mobilisation and altered insulin sensitivity (41, 42). It has been suggested in human studies that consumption of nutrients at inappropriate times of day results in metabolic imbalances as a result of circadian desynchrony (37). In our present study, participants were sleep deprived in both sessions, and melatonin onset was not significantly different between BL and DL, indicating that postprandial changes obtained are unlikely to be due to sleep deprivation or circadian misalignment. The difference observed in glucose tolerance and insulin sensitivity could be due to the presence of melatonin in the DL session, as melatonin has been reported to inhibit insulin secretion in both rat insulinoma cells and pancreatic islets, thus influencing blood glucose (43, 44). Furthermore, light exposure during sleep deprivation in humans has been reported to increase insulin resistance compared to sleep deprivation in the darkness (45). The explanation being possible due to dysregulation of the SCN that coordinates peripheral organs and energy homeostasis, in addition to altered melatonin levels, which have been associated with increased insulin resistance in experimental animals (46), while melatonin secretion was inversely correlated with insulin in healthy humans (47). An increase in evening light exposure and a decrease in urinary 6-sulfatoxymelatonin excretion have been associated with an increase in the prevalence of type 2 diabetes in elderly individuals (48). However, the effects of evening light exposure on glucose metabolism may be greater in the young compared to an older population due to reduced transmission of light through the lenses (49).

In contrast, a human study by Rubio-Sastre and coworkers gave exogenous melatonin prior to an oral glucose tolerance test (OGTT) in the morning and evening and resulted in raised insulin and glucose responses compared to placebo (17). This study conflicts with our results; however, this may be due to differences between the two study protocols. We utilised endogenous melatonin and a consumption of an evening meal, whereas Rubio-Sastre and coworkers administered a single dose of immediate-release melatonin and a drink of oral glucose. The sleep–wake cycle of participants in the Rubio-Sastre study was not recorded, which could influence metabolite changes. Using a single dose of immediate-release melatonin would saturate melatonin receptors with supraphysiological melatonin levels. Variability of absorption rates in oral glucose tolerance tests (OGTT) compared to our study that provide a standard evening meal could contribute to the result differences (17).

Pre-prandial plasma NEFA was significantly elevated in the DL session, which may be due to the physiological response to fasting itself. However, all participants had the same fasting period between lunch and the test meal during both sessions, and the main significant difference was observed just prior to the test meal (*T* = 0) when melatonin levels were already high in the DL. This potentially suggests the possible stimulatory effect of melatonin on glucagon (42). Other potential mechanism could be increased sympathetic action due to endogenous melatonin inducing HSL activity (50, 51, 52). It is suggested that the major activating factor for HSL is the absence of the inhibitory effects of insulin (53). No difference in plasma TAGs could be due to the absence of melatonin effects on LPL activity. Also, it is important to note that TAG levels normally take approximately 9 h to return to basal levels after a meal (54, 55, 56).

One of the limitation of this study was that postprandial response was only measured for up to 5 h after the standard evening meal, which does not provide a complete profile of postprandial TAGs. Future research needs to include a longer sampling period. Further hormonal analyses such glucagon and cortisol would be interesting to determine if NEFA changes were due to glucagon effects and to determine the role of cortisol in metabolic changes of glucose and insulin. The protocol used in this study can only explain changes due to different light sessions or endogenous melatonin action. A future study involving exogenous melatonin administration during light exposure would help to determine whether the metabolic changes seen are due to melatonin or other processes.

In conclusion, this is the first study to assess the influence of bright light exposure (room light) at night on metabolic and hormonal responses in healthy young participants. Significantly higher glucose and insulin in the BL session suggests glucose intolerance and insulin insensitivity. Elevated NEFAs level in the DL session prior to the meal could either be due to the stimulatory effects of melatonin on glucagon or the inhibitory effects on insulin, resulting in higher HSL activity. Our recent results could be due to light or melatonin or a combination of the two. These results support the idea that nocturnal lifestyle, such as in night shift work, is likely to be one of the risk factors to health in modern society, including diabetes. Further studies are needed to determine whether melatonin causes the present metabolic changes or other processes are involved.

## Declaration of interest

The authors declare that there is no conflict of interest that could be perceived as prejudicing the impartiality of the research reported.

## Funding

This research was supported by Abu Dhabi Health Service Company (SEHA) in United Arab Emirates.

## Authors’ contribution statement

The authors’ responsibilities were as follows: M S A, B M and S M H designed research; M S A conducted research; M S A analysed data; M S A wrote the paper; M S A, B M and S M H had primary responsibility for final content. All authors read and approved the final manuscript.

## References

[1] Fonken LK Nelson RJ. Illuminating the deleterious effects of light at night. F1000 Medicine Reports 2011 3 1–7. (10.3410/M3-18) 21941596 PMC3169904

[2] Navara KJ Nelson RJ. The dark side of light at night: physiological, epidemiological, and ecological consequences. Journal of Pineal Research 2007 43 215–224. (10.1111/j.1600-079X.2007.00473.x) 17803517

[3] LeGates TA Fernandez DC Hattar S. Light as a central modulator of circadian rhythms, sleep and affect. Nature Reviews Neuroscience 2014 15 443–454. (10.1038/nrn3743) 24917305 PMC4254760

[4] Kalsbeek A Fliers E Romijn J La Fleur S Wortel J Bakker O Endert E Buijs R. The suprachiasmatic nucleus generates the diurnal changes in plasma leptin levels. Endocrinology 2001 142 2677–2685. (10.1210/endo.142.6.8197) 11356719

[5] Kalsbeek A Ruiter M La Fleur SE Van Heijningen C Buijs RM. The diurnal modulation of hormonal responses in the rat varies with different stimuli. Journal of Neuroendocrinology 2003 15 1144–1155. (10.1111/j.1365-2826.2003.01112.x) 14636176

[6] Ruiter M, La Fleur SE, van Heijningen C, van der Vliet J, Kalsbeek A & Buijs RM. The daily rhythm in plasma glucagon concentrations in the rat is modulated by the biological clock and by feeding behavior. Diabetes 2003 52 1709–1715. (10.2337/diabetes.52.7.1709) 12829637

[7] Fukuda H & Iritani N. Diurnal variations of lipogenic enzyme mRNA quantities in rat liver. Biochimica et Biophysica Acta (BBA)-Lipids and Lipid Metabolism 1991 1086 261–264. (10.1016/0005-2760(91)90168-h) 1683792

[8] Yang X Downes M Ruth TY Bookout AL He W Straume M Mangelsdorf DJ Evans RM. Nuclear receptor expression links the circadian clock to metabolism. Cell 2006 126 801–810. (10.1016/j.cell.2006.06.050) 16923398

[9] Vinogradova IA Anisimov VN Bukalev AV Semenchenko AV Zabezhinski MA. Circadian disruption induced by light-at-night accelerates aging and promotes tumorigenesis in rats. Aging 2009 1 855 (10.18632/aging.100092) 20157558 PMC2816394

[10] Ekmekcioglu C Touitou Y. Chronobiological aspects of food intake and metabolism and their relevance on energy balance and weight regulation. Obesity Reviews 2011 12 14–25. (10.1111/j.1467-789X.2010.00716.x) 20122134

[11] Scheer F Ter Horst G van Der Vliet J Buijs R. Physiological and anatomic evidence for regulation of the heart by suprachiasmatic nucleus in rats. American Journal of Physiology: Heart and Circulatory Physiology 2001 280 H1391–H1399. 11179089 10.1152/ajpheart.2001.280.3.H1391

[12] Scheer FA Kalsbeek A Buijs RM. Cardiovascular control by the suprachiasmatic nucleus: neural and neuroendocrine mechanisms in human and rat. Biological Chemistry 2003 384 697–709. (10.1515/bc.2003.078) 12817466

[13] Wyse C Selman C Page M Coogan A Hazlerigg D. Circadian desynchrony and metabolic dysfunction; did light pollution make us fat? Medical Hypotheses 2011 77 1139–1144. (10.1016/j.mehy.2011.09.023) 21983352

[14] Arasteh A Aliyev A Khamnei S Delazar A Mesgari M Mehmannavaz Y. Investigation of the effects of constant darkness and light on blood serum cholesterol, insulin and glucose levels in healthy male rats. African Journal of Biotechnology 2013 9 6791–6796. (10.5897/AJB10.773)

[15] Zhang J Kaasik K Blackburn MR Lee CC. Constant darkness is a circadian metabolic signal in mammals. Nature 2006 439 340–343. (10.1038/nature04368) 16421573

[16] Peschke E Bähr I Mühlbauer E. Melatonin and pancreatic islets: interrelationships between melatonin, insulin and glucagon. International Journal of Molecular Sciences 2013 14 6981–7015. (10.3390/ijms14046981) 23535335 PMC3645673

[17] Rubio-Sastre P Scheer FA Gómez-Abellán P Madrid JA Garaulet M. Acute melatonin administration in humans impairs glucose tolerance in both the morning and evening. Sleep 2014 37 1715 (10.5665/sleep.4088) 25197811 PMC4173928

[18] Nishida S Sato R Murai I Nakagawa S. Effect of pinealectomy on plasma levels of insulin and leptin and on hepatic lipids in type 2 diabetic rats. Journal of Pineal Research 2003 35 251–256. (10.1034/j.1600-079X.2003.00083.x) 14521630

[19] Chan T Tang P. Effect of melatonin on the maintenance of cholesterol homeostasis in the rat. Endocrine Research 1995 21 681–696. (10.1080/07435809509030483) 7588436

[20] Kozirog M Poliwczak AR Duchnowicz P Koter-Michalak M Sikora J Broncel M. Melatonin treatment improves blood pressure, lipid profile, and parameters of oxidative stress in patients with metabolic syndrome. Journal of Pineal Research 2011 50 261–266. (10.1111/j.1600-079X.2010.00835.x) 21138476

[21] Wakatsuki A, Okatani Y, Ikenoue N, Kaneda C & Fukaya T. Effects of short-term melatonin administration on lipoprotein metabolism in normolipidemic postmenopausal women. Maturitas 2001 38 171–177. (10.1016/s0378-5122(00)00221-8) 11306206

[22] Kraemer FB Shen W-J. Hormone-sensitive lipase control of intracellular tri-(di-) acylglycerol and cholesteryl ester hydrolysis. Journal of Lipid Research 2002 43 1585–1594. (10.1194/jlr.R200009-JLR200) 12364542

[23] Wehrens SM Hampton SM Finn RE Skene DJ. Effect of total sleep deprivation on postprandial metabolic and insulin responses in shift workers and non-shift workers. Journal of Endocrinology 2010 206 205–215. (10.1677/JOE-10-0077) 20479040

[24] Scheer FA Hilton MF Mantzoros CS Shea SA. Adverse metabolic and cardiovascular consequences of circadian misalignment. PNAS 2009 106 4453–4458. (10.1073/pnas.0808180106) 19255424 PMC2657421

[25] Arendt J Bojkowski C Franey C Wright J Marks V. Immunoassay of 6-hydroxymelatonin sulfate in human plasma and urine: abolition of the urinary 24-hour rhythm with atenolol. Journal of Clinical Endocrinology and Metabolism 1985 60 1166–1173. (10.1210/jcem-60-6-1166) 3998065

[26] Arendt J Aldhous M Wright J. Synchronisation of a disturbed sleep-wake cycle in a blind man by melatonin treatment. Lancet 1988 331 772–773. (10.1016/S0140-6736(88)91586-3) 2895305

[27] Levy JC Matthews DR Hermans MP. Correct homeostasis model assessment (HOMA) evaluation uses the computer program. Diabetes Care 1998 21 2191 (10.2337/diacare.21.12.2191) 9839117

[28] Caumo A Bergman RN Cobelli C. Insulin sensitivity from meal tolerance tests in normal subjects: a minimal model index. Journal of Clinical Endocrinology and Metabolism 2000 85 4396–4402. (10.1210/jcem.85.11.6982) 11095485

[29] Coomans CP van den Berg SA Houben T van Klinken J-B van den Berg R Pronk AC Havekes LM Romijn JA van Dijk KW Biermasz NR. Detrimental effects of constant light exposure and high-fat diet on circadian energy metabolism and insulin sensitivity. Journal of the Federation of American Societies for Experimental Biology 2013 27 1721–1732. (10.1096/fj.12-210898) 23303208

[30] Mazepa R Cuevas M Collado P Gonzalez-Gallego J. Melatonin increases muscle and liver glycogen content in nonexercised and exercised rats. Life Sciences 1999 66 153–160. (10.1016/S0024-3205(99)00573-1) 10666011

[31] Gooley JJ Chamberlain K Smith KA Khalsa SBS Rajaratnam SM Van Reen E Zeitzer JM Czeisler CA Lockley SW. Exposure to room light before bedtime suppresses melatonin onset and shortens melatonin duration in humans. Journal of Clinical Endocrinology and Metabolism 2010 96 E463–E472. (10.1210/jc.2010-2098) 21193540 PMC3047226

[32] Cain SW Dennison CF Zeitzer JM Guzik AM Khalsa SBS Santhi N Schoen MW Czeisler CA Duffy JF. Sex differences in phase angle of entrainment and melatonin amplitude in humans. Journal of Biological Rhythms 2010 25 288–296. (10.1177/0748730410374943) 20679498 PMC3792014

[33] Fonken LK Workman JL Walton JC Weil ZM Morris JS Haim A Nelson RJ. Light at night increases body mass by shifting the time of food intake. PNAS 2010 107 18664–18669. (10.1073/pnas.1008734107) 20937863 PMC2972983

[34] Jha PK Challet E Kalsbeek A. Circadian rhythms in glucose and lipid metabolism in nocturnal and diurnal mammals. Molecular and Cellular Endocrinology 2015 418 74–88. (10.1016/j.mce.2015.01.024) 25662277

[35] Spiegel K Leproult R Van Cauter E. Impact of sleep debt on metabolic and endocrine function. Lancet 1999 354 1435–1439. (10.1016/S0140-6736(99)01376-8) 10543671

[36] Saper CB Scammell TE Lu J. Hypothalamic regulation of sleep and circadian rhythms. Nature 2005 437 1257–1263. (10.1038/nature04284) 16251950

[37] Bailey SM Udoh US Young ME. Circadian regulation of metabolism. Journal of Endocrinology 2014 222 R75–R96. (10.1530/JOE-14-0200) 24928941 PMC4109003

[38] Kreier F Kap YS Mettenleiter TC van Heijningen C van der Vliet J Kalsbeek A Sauerwein HP Fliers E Romijn JA Buijs RM. Tracing from fat tissue, liver, and pancreas: a neuroanatomical framework for the role of the brain in type 2 diabetes. Endocrinology 2006 147 1140–1147. (10.1210/en.2005-0667) 16339209

[39] Stamatakis KA Punjabi NM. EFfects of sleep fragmentation on glucose metabolism in normal subjects. Chest 2010 137 95–101. (10.1378/chest.09-0791) 19542260 PMC2803120

[40] Van Cauter E Knutson K Leproult R Spiegel K. The impact of sleep deprivation on hormones and metabolism. Medscape Neurology 2005 7 (available at: http://www.medscape.org/viewarticle/502825).

[41] Shiuchi T Haque MS Okamoto S Inoue T Kageyama H Lee S Toda C Suzuki A Bachman ES Kim Y-B. Hypothalamic orexin stimulates feeding-associated glucose utilization in skeletal muscle via sympathetic nervous system. Cell Metabolism 2009 10 466–480. (10.1016/j.cmet.2009.09.013) 19945404

[42] Yi C-X Serlie MJ Ackermans MT Foppen E Buijs RM Sauerwein HP Fliers E Kalsbeek A. A major role for perifornical orexin neurons in the control of glucose metabolism in rats. Diabetes 2009 58 1998–2005. (10.2337/db09-0385) 19592616 PMC2731521

[43] Peschke E Mühlbauer E Mußhoff U Csernus VJ Chankiewitz E Peschke D. Receptor (MT1) mediated influence of melatonin on cAMP concentration and insulin secretion of rat insulinoma cells INS-1. Journal of Pineal Research 2002 33 63–71. (10.1034/j.1600-079X.2002.02919.x) 12153439

[44] Picinato MC Haber EP Cipolla-Neto J Curi R De Oliveira Carvalho CR Carpinelli AR. Melatonin inhibits insulin secretion and decreases PKA levels without interfering with glucose metabolism in rat pancreatic islets. Journal of Pineal Research 2002 33 156–160. (10.1034/j.1600-079X.2002.02903.x) 12220330

[45] Gil-Lozano M Hunter PM Behan L-A Gladanac B Casper RF Brubaker PL. Short-term sleep deprivation with nocturnal light exposure alters time-dependent glucagon-like peptide-1 and insulin secretion in male volunteers. American Journal of Physiology: Endocrinology and Metabolism 2016 310 E41–E50. (10.1152/ajpendo.00298.2015) 26530153

[46] Cipolla-Neto J Amaral F Afeche S Tan D Reiter R. Melatonin, energy metabolism, and obesity: a review. Journal of Pineal Research 2014 56 371–381. (10.1111/jpi.12137) 24654916

[47] McMullan CJ Curhan GC Schernhammer ES Forman JP. Association of nocturnal melatonin secretion with insulin resistance in nondiabetic young women. American Journal of Epidemiology 2013 178 231–238. (10.1093/aje/kws470) 23813704 PMC3937598

[48] Obayashi K Saeki K Iwamoto J Ikada Y Kurumatani N. Independent associations of exposure to evening light and nocturnal urinary melatonin excretion with diabetes in the elderly. Chronobiology International 2014 31 394–400. (10.3109/07420528.2013.864299) 24328728

[49] Turner PL Van Someren EJ Mainster MA. The role of environmental light in sleep and health: effects of ocular aging and cataract surgery. Sleep Medicine Reviews 2010 14 269–280. (10.1016/j.smrv.2009.11.002) 20056462

[50] Song CK Bartness TJ. CNS sympathetic outflow neurons to white fat that express MEL receptors may mediate seasonal adiposity. American Journal of Physiology: Regulatory, Integrative and Comparative Physiology 2001 281 R666–R672. 10.1152/ajpregu.2001.281.2.R666 11448873

[51] Holm C, Østerlund T, Laurell H & Contreras JA. Molecular mechanisms regulating hormone-sensitive lipase and lipolysis. Annual Review of Nutrition 2000 20 365–393. (10.1146/annurev.nutr.20.1.365) 10940339

[52] Langin D Lucas S Lafontan M. Millennium fat-cell lipolysis reveals unsuspected novel tracks. Hormone and Metabolic Research 1999 32 443–452. (10.1055/s-2007-978670) 11246809

[53] Griffin BA Cunnane SC. Nutrition and metabolism of lipids. In Introduction to Human Nutrition, pp 86–121. Hoboken, NJ, USA: Wiley-Blackwell 2009.

[54] DeFronzo RA. The triumvirate: β-cell, muscle, liver. A collusion responsible for NIDDM. Diabetes 1988 37 667–687. (10.2337/diab.37.6.667) 3289989

[55] Ribeiro D Hampton S Morgan L Deacon S Arendt J. Altered postprandial hormone and metabolic responses in a simulated shift work environment. Journal of Endocrinology 1998 158 305–310. (10.1677/joe.0.1580305) 9846159

[56] Sopowski M Hampton S Ribeiro D Morgan L Arendt J. Postprandial triacylglycerol responses in simulated night and day shift: gender differences. Journal of Biological Rhythms 2001 16 272–276. (10.1177/074873001129001881) 11407787

